# Zebrafish Infection: From Pathogenesis to Cell Biology

**DOI:** 10.1016/j.tcb.2017.10.002

**Published:** 2018-02

**Authors:** Vincenzo Torraca, Serge Mostowy

**Affiliations:** 1 Section of Microbiology, MRC Centre for Molecular Bacteriology and Infection, Imperial College London, London, UK

**Keywords:** autophagy, bacterial infection, cellular microbiology, inflammation, innate immunity, zebrafish

## Abstract

The study of host–pathogen interactions has illuminated fundamental research avenues in both infection and cell biology. Zebrafish (*Danio rerio*) larvae are genetically tractable, optically accessible, and present a fully functional innate immune system with macrophages and neutrophils that mimic their mammalian counterparts. A wide variety of pathogenic bacteria have been investigated using zebrafish models, providing unprecedented resolution of the cellular response to infection *in vivo*. In this review, we illustrate how zebrafish models have contributed to our understanding of cellular microbiology by providing an *in vivo* platform to study host–pathogen interactions from the single cell to whole animal level. We also highlight discoveries made from zebrafish infection that hold great promise for translation into novel therapies for humans.

## Trends

Due to genetic and physiological similarities with higher vertebrates including humans, zebrafish are useful to study infection from a wide variety of bacterial pathogens.

A vast array of genetic, optical, and chemical tools applicable to zebrafish provide an innovative toolkit to investigate cellular microbiology *in vivo*.

The study of zebrafish infection is providing unprecedented resolution of host–pathogen interactions, and discovering novel mechanisms of cellular immunity.

The zebrafish can be used as a platform to develop therapeutic strategies for humans suffering from disease related to infection and inflammation.

## Cell Biology: Zebrafish Take the Stage

**Figure 1:**
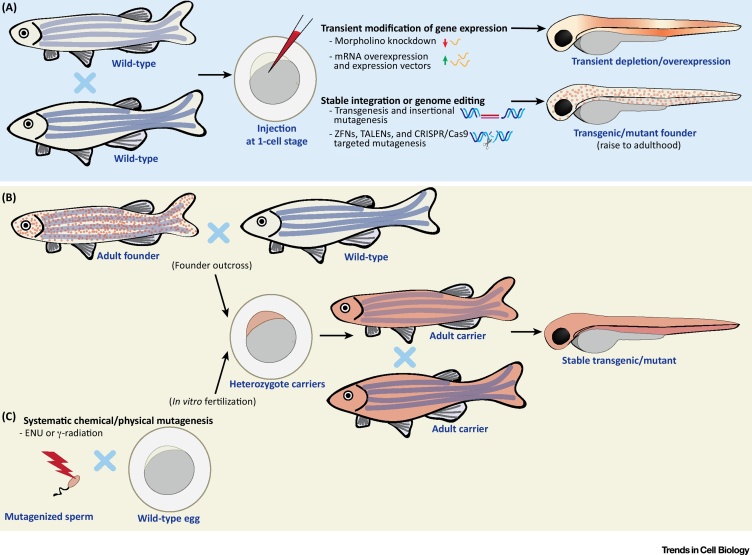
Approaches for Genetic Manipulation of Zebrafish. (A) Injection of constructs and chemicals in zebrafish eggs. Transient depletion can be performed by injection of morpholino oligonucleotides, RNA-binding oligomers that block translation/maturation of a specific (pre)-mRNA. Morpholinos can sometimes elicit off-target effects, therefore, it is important to validate phenotypes using alternative strategies and/or rescue experiments before conclusions can be fully drawn. Transient expression of genes can be obtained by injection of synthesized mRNA or plasmid DNA bearing an expression construct. Injected mRNAs will be expressed ubiquitously, while injection of plasmids enables cell- or tissue-specific expression. Zebrafish eggs can stably integrate DNA, which can be used to obtain stable transgenic lines or insertional mutants. The frequency of transgenesis is low when injecting DNA alone, but can be increased using transposases (i.e., Tol2) or meganucleases (i.e., I-SceI meganuclease). Zebrafish stable mutants can be efficiently generated with ZFNs, TALENs, or CRISPR/Cas9. These systems are based on induction of a site-specific double-stranded break, which is repaired via an error-prone non-homologous end joining mechanism. The CRISPR/Cas9 system has recently become the most common method to generate zebrafish mutants. Additionally, the CRISPR/Cas9 system has also been adapted to generate conditional/tissue-specific knockouts. Mutants are obtained by injecting mRNA or protein for the nuclease (together with guide RNA in the case of CRISPR/Cas9) in zebrafish eggs. Conditional/tissue-specific mutants are obtained by integration of a construct where Cas9 expression is controlled by an inducible or tissue-specific promoter. DNA constructs for stable integration can be designed with flanking homology recombination arms, which drive integration into a precise locus, and allow generation of knock-in lines. Generation of precise knock-in zebrafish is still challenging but can be facilitated by introducing double strand breaks at the site of interest (i.e., using TALENs or CRISPR/Cas9) [6]. Embryos manipulated using these techniques can be used for downstream functional studies, or, in the case of stable modifications, be raised to adulthood to establish a novel line. (B) Selection of stable transgenic or mutant lines. Carriers from (A) can be outcrossed to obtain heterozygote carriers. These offspring can be used for experiments, or raised to adulthood and inbred (i.e., to obtain homozygotes). (C) Systematic mutagenesis. Large libraries of random mutations can be obtained by exposure of sperm to chemical or physical mutagens (i.e., ENU or γ-radiation) prior to *in vitro* fertilization. Abbreviations: CRISPR/Cas9, clustered regulatory interspaced short palindromic repeats/CRISPR associated protein 9; ENU, *N*-ethyl *N*-nitrosurea; TALEN, transcription activator-like effector nuclease; ZFN, zinc-finger nuclease.

The transparency of zebrafish embryos first attracted the attention of developmental biologists almost a century ago [1]. Zebrafish embryogenesis is rapid, *ex utero*, and amenable to noninvasive intravital imaging and longitudinal analysis *in vivo*. In the 1980s, research using zebrafish exploded as their genetic and chemical tractability was discovered (Figure 1), features at the time unprecedented for a vertebrate model 2, 3, 4, 5. The combination of optical accessibility and genetic tractability allows researchers to label proteins and cell types for high-resolution fluorescent microscopy. The zebrafish also enables the characterization of gene function via overexpression, transient depletion, or genome editing [6]. The ease of obtaining hundreds of embryos combined with the possibility of pharmacological treatment via bath water exposure allows high throughput analyses, a feature previously available only for tissue culture cells or invertebrate species. The zebrafish genome has been fully sequenced, highlighting a remarkable similarity with humans [7]. At least 70% of the human coding genome, including genes associated with disease, have a direct ortholog in zebrafish. Collectively, zebrafish embryos and larvae are widely accepted in the 21^st^ century as *in vivo* models for a variety of human diseases including cancer, inflammatory disorders, and infection.

**Figure 2:**
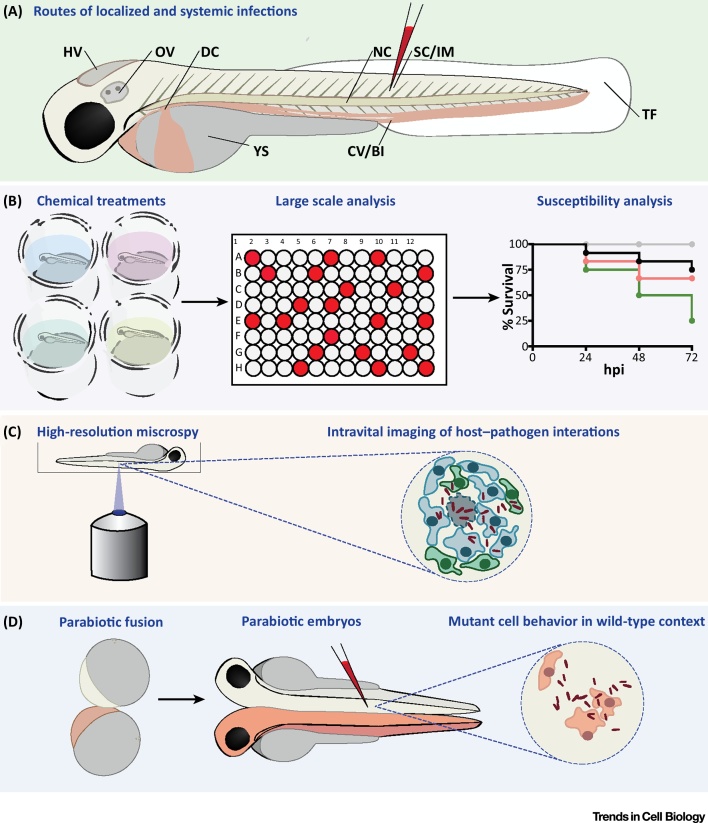
Methods for Studying Host–Pathogen Interactions Using Zebrafish. (A) Routes of zebrafish injection. Larvae can be injected locally into the YS or in body cavities, such as the HV and OV. Other compartments for injection include SC, IM, or the NC. HV, OV, IM, and TF infection all permit study of immune cell recruitment. The NC is inaccessible to immune cells but is valuable to model bone and cartilage inflammation. Injection into the circulation can be achieved by intravenous injections, for example via the CV/BI or the DC. This results in a rapid systemic dissemination of microbes throughout the body. (B) Chemical treatments. Zebrafish are suitable for toxicology research and for screening of libraries of bioactive compounds, including antimicrobials, because molecules in the bath water can be absorbed via the zebrafish skin. Survival and bacterial burden can be quantified to compare susceptibility of different genetic conditions or to assess the effect of chemicals/therapeutics in disease prevention. (C) Intravital imaging. Host–pathogen interactions can be followed *in vivo* by combining fluorescently-labeled bacteria and zebrafish transgenic lines reporting the expression pattern of specific genes or labeling specific cell types. A variety of proteins and subcellular compartments can also be tagged by fusing specific markers with fluorescent tags. (D) Parabiosis. Two zebrafish embryos can be fused by surgically forcing their blastulae into direct contact. This results in the development of conjoined embryos sharing blood circulation and body parts, enabling secreted factors and circulating cells to distribute in the bodies of both individuals. When applied to embryos with different genetic/transgenic makeup, this technique is useful to distinguish cell-autonomous from non-cell-autonomous functions. Abbreviations: BI, blood island; CV, caudal vein; DC, duct of Cuvier; HV, hindbrain ventricle; IM, intramuscular; NC, notochord; OV, otic vesicle; SC, subcutaneous; TF, tail fin; YS, yolk sac.

The zebrafish model entered the field of host–pathogen interactions in 1999, when Philippe Herbomel and colleagues described **primitive macrophages** (see Glossary) emerging in the developing embryo from 22 hours postfertilization (hpf) [8]. These cells emulate primitive macrophages that defend mammalian embryos, and were observed to clear intravenously injected Gram-negative (*Escherichia coli*) and Gram-positive (*Bacillus subtilis*) bacteria. From approximately 36 hpf, primitive neutrophils appear and synergize with macrophages for host defense 9, 10. Due to the early emergence of innate immune cells and the ability to perform both local and systemic infections via microinjection (Figure 2), zebrafish infection models have been established for numerous bacterial, viral, and fungal pathogens 11, 12, 13. Instead of covering the whole field of zebrafish–microbe interactions, here we focus on recent studies where infection of zebrafish larvae with bacterial pathogens has significantly advanced our understanding of both pathogenesis and cell biology. To highlight the breadth of zebrafish infection models currently available, we provide examples of infection using Gram-negative bacteria (*Salmonella* Typhimurium, *Shigella flexneri*, *Pseudomonas aeruginosa*, and *Burkholderia cenocepacia*), Gram-positive bacteria (*Listeria monocytogenes* and *Staphylococcus aureus*), and mycobacteria (*Mycobacterium marinum*, *Mycobacterium abscessus*, and *Mycobacterium leprae*).

## *Salmonella* Typhimurium: New Links between Metabolism and Inflammation

*S. enterica* serovar Typhimurium is an important **zoonotic** pathogen causing gastroenteritis and inflammation of the intestinal mucosa. Injection of *S.* Typhimurium into zebrafish leads to a typhoid-like disease caused by *S.* Typhimurium in mice and *S. enterica* serovar Typhi in humans. Inflammation is a key determinant of *Salmonella* pathogenesis, however, the molecular mechanisms that regulate inflammation during infection are not fully understood. New work has reported that zebrafish guanylate-binding protein 4 (Gbp4) is required for clearance of *S.* Typhimurium via activation of the **inflammasome** in neutrophils [14]. In this case, neutrophils are recruited to the infected tissue by local release of leukotriene B4, an inflammatory lipid mediator, which synergizes with C-X-C motif chemokine ligand 8 (Cxcl8) for neutrophil **chemotaxis**. The recruited neutrophils engulf *Salmonella* and activate the Gbp4 inflammasome, which modulates activity of cytosolic phospholipase A2 and production of **prostaglandins**, supporting clearance of infection. Together, these data suggest that activation of the inflammasome can be used to treat bacterial disease, and show an important role for neutrophils in mediating this response *in vivo*.

**Figure 3:**
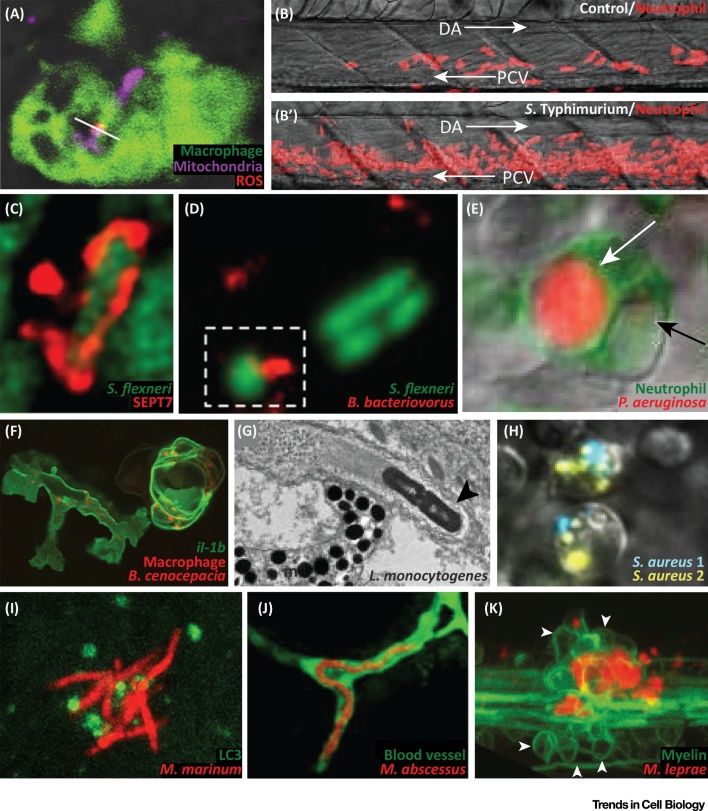
Key Figure: Hallmarks of Bacterial Infection Observed Using Zebrafish

Landmark studies have suggested that reactive oxygen species (ROS), produced within mitochondria during oxidative phosphorylation, are crucial for killing intracellular bacterial pathogens [15]. Work has shown that the mitochondria-localizing enzyme encoded by immunoresponsive gene 1 (*irg1*) is induced in macrophages during *Salmonella* infection of zebrafish [16]. The depletion of *irg1* from macrophages prevents the ability to fuel oxidative phosphorylation with fatty acids, significantly reducing the production of mitochondria-derived ROS and bactericidal activity (Figure 3A, Key Figure). Similar observations have been made using lipopolysaccharide (LPS)-activated bone-marrow-derived macrophages, where *Irg1* induction modulates mitochondrial respiration and leads to a metabolic shift affecting production of inflammatory mediators and macrophage effector function [17]. These findings uncover a new link between metabolic reprogramming and the production of immune effectors, highlighting an important role for metabolism in host defense [18].

Considering that neutrophils are key for host defense but are generally short lived, **emergency granulopoiesis** is required for the replenishment of neutrophils consumed during infection [19]. Using a *Salmonella*–zebrafish infection model, it has been shown that increased neutrophil production during infection is a direct consequence of **hematopoietic stem and progenitor cell** (**HSPC**) expansion [20] (Figure 3B,B′). Mechanistically, this expansion is dependent on the production of granulocyte colony-stimulating factor (G-CSF) by macrophages, to which HSPCs respond by upregulating inducible nitric oxide synthase (iNOS). iNOS regulates the urea cycle to produce NO, a pleiotropic signaling mediator that activates HSPC proliferation. Induction of iNOS is promoted by CCAAT/enhancer-binding protein β (C/EBPβ), a transcription factor also implicated in G-CSF-dependent emergency granulopoiesis in mammals [21]. These results describe a novel HSPC response to infection, and uncover signaling pathways that can be manipulated to fight infection and inflammatory stress.

Taken together, *Salmonella* infection of zebrafish has been key for discovery of novel concepts in cell-autonomous immunity, immunometabolism, and emergency granulopoiesis. Moreover, these studies reveal fundamental innate immune pathways for innovative therapeutic interventions, relevant for a wide variety of bacterial infections.

##  *Shigella flexneri*: A Versatile Infection Paradigm to Study Inflammation *In Vivo*

*Shigella* is human pathovar of *E. coli* causing gastrointestinal infections and bacillary dysentery, a disease characterized by bloody diarrhea. Although no nonprimate animal model exists that closely mimics shigellosis, a variety of steps underlying the *Shigella* infection process can be examined using zebrafish. When injected into zebrafish, *Shigella* can invade epithelial cells and induce hallmarks of human shigellosis, including inflammation and macrophage cell death 22, 23. Infection of zebrafish by *Shigella* is strictly dependent on the **type 3 secretion system** (**T3SS**), a bacterial determinant essential for human disease. Neutrophils are important for host defense against *Shigella*, and phagocytose bacteria and dying cells that fail to control infection. As observed from infection of tissue culture cells and zebrafish, intracellular *Shigella* can escape to the cytosol and be targeted to **autophagy** 22, 24. To restrict bacterial replication, actin-polymerizing bacteria targeted to autophagy are entrapped in **septin cages** (Figure 3C). At the whole animal level, the depletion (via morpholino targeting the autophagy receptor p62) or stimulation (via the mammalian target of rapamycin inhibitor rapamycin) of autophagy both result in increased bacterial burden and host susceptibility to infection [22]. In agreement with the breadth of roles described for autophagy in cell biology [25], and also with recent literature studying bacterial autophagy using mice [26], these results indicate that autophagy needs to be carefully controlled *in vivo* to protect against bacterial infection.

The *Shigella–*zebrafish infection model has been useful to discover new roles for the cytoskeleton in cellular immunity [27]. Zebrafish septins have been implicated in the restriction of inflammation induced by *Shigella* [23]. The depletion of Sept15 (a zebrafish ortholog of mammalian Sept7, essential for septin function) during *Shigella* infection led to significantly increased activity of caspase-1 and host cell death, resulting in severe neutropenia and zebrafish killing. Inflammation induced by *Shigella* and Sept15 deficiency can be counteracted by treatment with anakinra, an anti-inflammatory drug that blocks interleukin-1β (IL-1β) signaling. These findings reveal that septins are important to restrict inflammatory signaling *in vivo*, and suggest that anakinra can be used as a therapy to restrict inflammation during infection.

To combat the rising incidence of antimicrobial resistance, improved and creative therapies are urgently needed. *Bdellovibrio bacteriovorus* is a predatory bacterial species gaining recognition for its ability to invade and kill other Gram-negative bacteria. Using a model of localized hindbrain infection with a multidrug-resistant strain of *S. flexneri*, it was discovered that *B. bacteriovorus* can work in synergy with the host innate immune system to eradicate an infection otherwise untreatable with first line antibiotics [28]. These findings serve as proof of principle that predatory bacteria can be used as a ‘living antibiotic’ *in vivo* (Figure 3D).

Collectively, the use of zebrafish to study *Shigella* infection has provided fundamental advances in our understanding of autophagy and inflammation, and was first to investigate the cell biology of bacterial predator–prey interactions *in vivo*. This information should provide vital clues for the development of new therapeutic strategies against *Shigella* and other multidrug-resistant infections.

## *Pseudomonas aeruginosa*: Pathogenic Mechanisms Relevant to Clinical Settings

*P. aeruginosa* is an opportunistic bacterium, commonly infecting the lungs of cystic fibrosis (CF) patients. Here, the inability of phagocytes to clear the airways facilitates establishment of persistent infection. The zebrafish is relatively resistant to *Pseudomonas*, and large inocula are required to establish infection and host killing 29, 30. As in humans, the depletion of phagocytes can dramatically increase the susceptibility of larvae to *P. aeruginosa* [29]. In agreement with a key role for phagocytes in *Pseudomonas* control, both macrophages and neutrophils can rapidly engulf and kill systemically delivered bacteria (Figure 3E). Notably, T3SS mutants are attenuated or virulent in wild-type or phagocyte-depleted hosts, respectively, indicating that *Pseudomonas* pathogenesis in immunocompetent hosts is mediated by T3SS–phagocyte interactions. Consistent with this, *Pseudomonas* T3SS mutants are attenuated at developmental stages when both macrophages and neutrophils are present, yet lethal at earlier stages when only macrophages have started to emerge [30].

In lungs of CF patients, the persistence of *P. aeruginosa* is associated with a lifestyle switch (from free floating to biofilm forming) enabled by **cyclic-di-GMP**. The diguanylate cyclase SadC plays a key role in controlling the levels of cyclic-di-GMP in *P. aeruginosa*, and the methyltransferase WarA was recently identified as an interacting partner of SadC [31]. Together SadC and WarA interact with the LPS biosynthesis machinery of *P. aeruginosa* to modify the distribution of LPS O antigen. SadC and WarA mutants are attenuated during zebrafish infection because the recruitment of neutrophils to mutant bacteria is significantly increased [31]. These results demonstrate a novel function for SadC/WarA modifications of LPS in mediating immune evasion *in vivo*.

Overall, the zebrafish has helped to elucidate mechanisms of pathogenesis underlying opportunistic *Pseudomonas* infection, and serves as a platform to reveal bacterial effectors required for escape from innate immunity. It can also be used to suggest novel therapies aimed at boosting innate immune function to control opportunistic bacterial infection in humans.

## *Burkholderia cenocepacia:* Inflammatory Macrophages as a Replicative Niche

Similar to *P. aeruginosa*, *B. cenocepacia* is a major health issue for CF patients. In the case of *B. cenocepacia* infection, primary colonization of the lungs by bacteria leads to **abscesses**, bacteremia, and the progressive decline of lung function (called cepacia syndrome). From work performed *in vitro*, it was widely believed that *B. cenocepacia* form extracellular biofilms in the lungs of CF patients, resembling those formed by *P. aeruginosa* 32, 33. However, recent work has challenged this, reporting that *Burkholderia* resides in phagocytes infiltrating the lungs [32]. To investigate *Burkholderia* pathogenesis *in vivo*, a *B. cenocepacia*–zebrafish infection model has been developed [34]. Work has shown that zebrafish larvae are highly susceptible to the ET12 strain of *B. cenocepacia* (a clinical isolate hypervirulent in humans and other animal models), but can tolerate infection from less virulent members of the *B. cepacia* complex, including strains of *Burkholderia vietnamiensis* and *Burkholderia stabilis*.

Although macrophages and neutrophils have been observed to interact with *Burkholderia* injected into zebrafish, macrophages are essential for *B. cenocepacia* survival and replication *in vivo* 33, 34. By contrast, neutrophils do not affect bacterial replication or disease outcome. Consistent with a crucial role for macrophages in *Burkholderia* infection, the chemical ablation of macrophages significantly reduced bacterial replication and host susceptibility to infection [33]. Intramacrophage replication of *Burkholderia* is fundamental for the transition from chronic to acute disease, characterized by inflammation and zebrafish death. In agreement with this, macrophages are a major source of Il-1β during *Burkholderia* infection (Figure 3F). However, the role of inflammation in mediating resistance or susceptibility to *Burkholderia* is not yet clear: depletion of Il-1β is probacterial, while modulation of Il-1β signaling with anakinra is antibacterial. Further application of the zebrafish model will be important to decipher *in vivo* the precise role of inflammation in *Burkholderia* pathogenesis.

Together, these results show that macrophages, and not neutrophils, are critical for *B. cenocepacia* replication and inflammation. Zebrafish infection therefore complements *in vitro* models and clinical studies, and can be used to investigate mechanisms underlying *Burkholderia* pathogenesis *in vivo*.

## *Listeria monocytogenes*: Recent Advances in Understanding Host Defense Against Cytosolic Bacteria

*L. monocytogenes* is a food-borne pathogen causing a variety of symptoms in infected humans, ranging from fever to life-threatening septicemia. Two major virulence factors of *Listeria* include lysteriolisin O (LLO), a pore forming toxin that enables escape from the internalization vacuole, and ActA, a bacterial mimic of the actin nucleation factor WASP (Wiskott–Aldrich syndrome protein), which polymerizes actin tails for evasion of cellular immunity. Injections of zebrafish have shown that blood-borne *Listeria* is rapidly engulfed by macrophages and neutrophils [35]. Both LLO-dependent vacuole escape and ActA-dependent actin tail formation can be observed in zebrafish, where LLO and ActA mutants are attenuated. These observations demonstrate *in vivo* relevance for hallmarks of *Listeria* infection previously described *in vitro* (Figure 3G).

A *L. monocytogenes* strain engineered to ectopically secrete monomers of *Legionella pneumophila* flagellin (called *Lm*-pyro) has been useful to demonstrate a role for inflammasome activation in host defense. It has been shown, both *in vitro* using bone-marrow-derived macrophages and *in vivo* using mice, that *Lm*-pyro activates the inflammasome and is attenuated (as compared to wild-type *Listeria*) [36]. Similarly, during infection of zebrafish, *Lm*-pyro can activate the inflammasome in macrophages, leading to attenuation *in vivo* [37]. In agreement with a role for the inflammasome in host defense, depletion of macrophages or of a zebrafish ortholog of caspase-1 restored virulence of the *Lm*-pyro strain.

How *Listeria* interacts with the plasma membrane of cells is not fully understood. GP96, an endoplasmic reticulum chaperone, is involved in plasma membrane blebbing upon exposure to LLO [38]. The formation of membrane blebs can act as mechanism of host defense, protecting against host cell lysis mediated by pore-forming toxins. Work using tissue culture cells and zebrafish showed that GP96 can interact with nonmuscle myosin heavy chain IIA and control bleb formation. As a result, GP96 has a key role in cytoskeletal organization, cell migration, and plasma membrane integrity. Consistent with GP96 playing an important role in protection from LLO-dependent killing, the depletion of Gp96 *in vivo* significantly reduced zebrafish survival during *Listeria* infection.

Collectively, infection of zebrafish has been useful to highlight *L. monocytogenes* as a paradigm for *in vivo* investigation of cellular microbiology and bacterium–phagocyte interactions. It can be predicted that future studies using *Listeria* infection of zebrafish will illuminate fundamental aspects of host defense against cytosolic bacteria.

## *Staphylococcus aureus*: Neutrophils as an Immunological Bottleneck

*S. aureus* is a common member of the skin and mucosal microflora in humans, but in hospitalized settings induce a variety of complications including abscesses, pneumonia, and **septicemia**. Abscesses and septicemia caused by *S. aureus* can also be observed in zebrafish via the injection of bacteria in the blood [39]. *S. aureus* is viewed as an extracellular pathogen, however, work using a *S. aureus*–zebrafish infection model has revealed an important intracellular life cycle for *S. aureus in vivo* [40]. Upon systemic infection of zebrafish, macrophages and neutrophils clear the bulk of injected *S. aureus*, yet few **persisters** that evade phagocyte killing remain viable and replicate (Figure 3H). This situation creates an immunological bottleneck, resulting in clonal selection. Neutrophils are the main replicative niche where the selection of bacterial clones occurs, and the depletion of neutrophils can significantly reduce clonal selection. *S. aureus* is notorious for development of antibiotic resistance, and a subcurative dose of antibiotics in zebrafish or mice can enable preferential expansion of antibiotic-resistant clones *in vivo* [41]. This phenomenon can be explained by the phagocyte-dependent clonal selection characteristic of *S. aureus* infection.

*S. aureus* infection of zebrafish has been instrumental to discover a role for nerve growth factor β (NGFβ) in innate immunity [42]. Innate immune factors are not always conserved from invertebrates to vertebrates, and the *Drosophila Toll* ligand *Spaetzle* was viewed to lack a vertebrate counterpart. However, NGFβ present in chordates displays structural similarities to *Drosophila Spaetzle*, and exerts a *Spaetzle*-like function in the immune response against *S. aureus*. Activation of NOD-like receptors (NLRs) by recognition of *S. aureus* exoproteins leads to release of NGFβ by macrophages and stimulates bacterial killing. Mutations in NGFβ, or its high-affinity catalytic receptor tropomyosin receptor kinase A (TRKA), are linked to increased severity of *S. aureus* infection in humans. Zebrafish depleted for *TrkA* also show increased susceptibility to *S. aureus* infection, supporting an evolutionarily conserved role for the NGFβ–TRKA axis in host defense.

In summary, *S. aureus* infection of zebrafish has revealed evolutionarily conserved components of antistaphylococcal immunity, as well as fundamental mechanistic insights into bacterial persistence. These results have broad implications for the design of novel therapeutic strategies that can effectively limit disease outcome and the selection of antimicrobial resistant strains.

## *Mycobacterium marinum*: An Architect of Immune Evasion

*M. marinum,* closely related to *Mycobacterium tuberculosis* (the causative agent of human tuberculosis), is a natural pathogen of aquatic species, including amoebae, invertebrates, amphibians, and fish [43]. As a result, *M. marinum* infection of zebrafish has been the subject of intense investigation and used to discover fundamental mechanisms underlying tuberculosis in humans 44, 45, 46. Pioneering work in zebrafish infected with *M. marinum* provided the first evidence that mycobacterial **granulomas** are initiated by macrophages in response to virulence determinant region of difference 1 (RD1), and do not strictly require an adaptive immune response 47, 48. While macrophages have an essential role in granuloma initiation, neutrophils do not. Instead, neutrophils are recruited to granuloma aggregates during advanced and inflammatory stages, where they participate in clearance of bacteria and debris from necrotic macrophages [49]. Inflammatory status is crucial for establishment of mycobacterial infection [50]. A forward genetic screen in zebrafish revealed a key role for proinflammatory enzyme leukotriene A4 hydrolase (Lta4h) in susceptibility to mycobacteria, a finding also observed in humans [51]. Moreover, levels of tumor necrosis factor (TNF) are important for mycobacterial restriction, as TNF controls production of macrophage ROS [52]. In this case, low or high levels of TNF/ROS in macrophages can compromise microbicidal activity or lead to cell death, respectively.

The role of macrophages during *M. marinum* infection is complex. The depletion of macrophages, dysregulation of myeloid growth factors, or engorgement of macrophages with undigested contents (such as in lysosomal-storage disorders) all result in failure to control bacterial replication *in vivo* 53, 54, 55. By contrast, the reduction of macrophage recruitment to the developing granuloma can limit bacterial dissemination and granuloma expansion 53, 56. In agreement with this, work using mouse models has shown that reduction (but not complete ablation) of macrophages is protective against *M. tuberculosis* infection [57]. The activation state of macrophages is crucial for mycobacterial control, underscored by the evolution of mycobacterial surface lipids that mask pathogen-associated molecular patterns to avoid macrophage recognition [58]. Tissue-resident macrophages are first to respond to infection and can eliminate bacteria. However, monocytes recruited from circulation fuse with infected macrophages and mycobacteria are transferred to a more permissive niche [59]. Monocyte recruitment depends on the infection of resident macrophages and requires recognition of bacterial phenolic glycolipids, cytosolic sensing via stimulator of interferon genes (STING), and expression of C-C motif chemokine ligand 2 (Ccl2), a potent monocyte chemoattractant.

Although *M. marinum* can avoid Toll-like receptor (TLR) recognition early during infection, TLR signaling is crucial to mediate antimycobacterial autophagy at later and more inflammatory stages [60] (Figure 3I). Macrophages use phagolysosome maturation to kill mycobacteria, however, *M. marinum* can slowly replicate in these compartments because of MarP, a virulence factor that enables acid tolerance [61]. Strikingly, in response to *M. marinum* infection, macrophages are reprogrammed to upregulate epithelial cell markers and undergo mesenchymal to epithelial transition characterized by the formation of tight junctions within the granuloma [62]. While this process can limit bacterial dissemination, it also impedes access to the granuloma core by newly recruited immune cells. Depending upon hypoxia induction, macrophages activate a proangiogenetic program to promote granuloma expansion and bacterial growth [63]. In agreement with this, antiangiogenic therapy has been shown to attenuate granuloma formation 63, 64.

In summary, infections with the natural fish pathogen *M. marinum* has provided a variety of novel insights into human tuberculosis, fundamentally revised our interpretation of tubercular granulomas, and has suggested new therapeutic avenues to counteract tuberculosis in humans. Moreover, *M. marinum* infection of zebrafish can be used to reflect the heterogeneity of pathogenesis observed in human tuberculosis patients, enabling *in vivo* evaluation of risk factors and personalized antimycobacterial drug regimens.

## *Mycobacterium abscessus:* Illuminating Mechanisms of Virulence for an Emerging Bacterial Threat

The *M. abscessus* complex is a group of fast-growing mycobacteria commonly associated with post-traumatic wound sepsis and infection of vulnerable hosts. Treatment is challenging, in part because *M. abscessus* is resistant to many antibiotics [65]. *M. abscessus* can transit from a smooth (S) morphotype expressing cell surface glycopeptidolipids to a rough (R) morphotype devoid of glycopeptidolipids and clinically more virulent. Using a zebrafish infection model, the pathogenicity of S versus R morphotypes was compared [66]. It was discovered that attenuation of the S morphotype can be attributed to its lack of **cording**, a feature essential for the R morphotype to prevent phagocytosis and initiate abscess formation (Figure 3J). A follow*-*up study identified a bacterial dehydratase (MAB_4780) as essential to mediate cording *in vivo* [67]. Genetic mutants for this enzyme could not form extracellular cords and are attenuated in both wild-type and immunocompromised larvae.

Activation of TNF signaling is required to control *M. abscessus* infection, and depletion of TNF receptor 1 (Tnfr1) can increase susceptibility to both S and R morphotypes [68]. In agreement with findings from *M. marinum* infection, the depletion of TNF signaling reduced the microbicidal activity of macrophages and the recruitment of neutrophils to foci of *M. abscessus* infection, leading to unrestricted bacterial growth. These observations are consistent with evidence showing that anti-TNF therapies promote *M. abscessus* infection in patients.

Together, these findings reveal bacterial cording as an important mechanism of immune evasion for *M. abscessus*, and suggest that inhibition of cording can prevent *M. abscessus* pathogenesis *in vivo*. Additionally, zebrafish infection has been useful to explain why immunosuppressive TNF therapy leads to the exacerbation of *M. abscessus* infection in humans.

## *Mycobacterium leprae*: A New Role for Macrophages in Leprosy

*M. leprae* is the causative agent of leprosy, a debilitating disease characterized by damage to the peripheral nervous system. Leprosy remains poorly understood because bacteria are noncultivable in axenic conditions, and animal models of infection are limited to the mouse foot pad or the nine-banded armadillo (*Dasypus novemcinctus*, a natural host of infection). Morbidity caused by *M. leprae* is mostly attributed to the ability of the pathogen to cause axon demyelination, resulting in peripheral neuropathy, yet how axon demyelination is triggered by *M. leprae* is not fully defined. When injected into zebrafish, *M. leprae* interacts with macrophages to cross the vascular endothelium and invade peripheral tissues, where it can form granulomas that resemble those induced by *M. marinum* [69]. *M. leprae*, but not *M. marinum*, can trigger demyelination and peripheral axon damage associated with the expression of *M. leprae* phenolic glycolipid-1 (PGL-1) (Figure 3K). In agreement with this, *M. marinum* engineered to express *M. leprae* PGL-1 also triggers demyelination *in vivo*. Remarkably, PGL-1 does not demyelinate axons *per se*. Instead, macrophages mediate neuronal damage, and *M. leprae* is unable to trigger demyelination in macrophage-depleted hosts. Using infection with *M. marinum* expressing *M. leprae* PGL-1, it was shown that macrophages upregulate iNOS for axonal damage, and treatment with iNOS inhibitors can be used to significantly reduce macrophage-induced axonal damage.

Strikingly, infection of zebrafish larvae with *M. leprae* represents a valuable system to study peripheral neuropathy (a hallmark of leprosy infection), and reveals a new role for macrophages and nitrosative stress in mediating disease outcome. The *M. leprae* model of zebrafish infection also highlights the potential of zebrafish to study host–pathogen interactions underlying neglected diseases.

## Concluding Remarks

We have illustrated how zebrafish models of bacterial infection can reveal key aspects of infection biology and provide fundamental advances in understanding the biology of cellular immunity. It is to be expected that the study of host–pathogen interactions using zebrafish will continue to illuminate the complexity that underlies bacterial infection in higher vertebrates, including humans.

Considering other animal models currently available, what is the future of zebrafish research in infection biology (see Outstanding Questions)? The full potential of zebrafish infection has yet to be realized, and the application of advanced gene editing and high-resolution microscopy techniques will further promote this model for better understanding pathogenesis and fundamental cellular processes. What determines the extent to which a zebrafish infection model is useful? Clearly, a key strength of the zebrafish-infection model lies in its versatility and enabling of rapid discovery. We predict these properties will be valuable to investigate the cell biology of emerging and neglected pathogens *in vivo*, and to discover unforeseen aspects of the host–pathogen interface. How can zebrafish infection be exploited for clinical application? Zebrafish models are increasingly used in preclinical drug development and toxicity testing, and work performed using *M. marinum* has been a premiere example of how zebrafish can be used to develop therapeutic interventions. For in-depth molecular understanding of mechanisms and pathways, it will be critical to complement *in vitro* models using tissue culture cells with *in vivo* models using zebrafish. Finally, a major issue will be to validate the molecular and cellular events discovered during zebrafish infection using higher vertebrate models, including mice. This information should also provide vital clues for the development of new therapeutic strategies against human infectious diseases.

Outstanding Questions Can we use zebrafish to rapidly obtain insight into the pathogenesis and cell biology of poorly characterized and neglected pathogens? Can zebrafish be efficiently used in developing countries to advance science and screen local infectious diseases?

Can we generate humanized zebrafish to recapitulate highly specialized host–pathogen processes underlying human infections?

How will zebrafish contribute to emerging concepts such as bacterial persistence, epigenetics of host–pathogen interactions, roles of microbiota in health and disease, and trained innate immunity?

How will genetic screens and functional studies performed in zebrafish illuminate heterogeneity in the host response to bacterial infection? Can zebrafish help to personalize infection treatments in humans?

To what extent can zebrafish infection models guide mammalian studies, and vice versa?

How can zebrafish infection models be exploited for therapeutics and clinical application?

## Glossary

**Abscess**: local collection of purulent fluids frequently caused by pyogenic bacteria.
**Autophagy**: highly conserved intracellular degradation process involving membrane compartmentalization of a cytosolic substrate targeted to lysosomal delivery.
**Chemotaxis**: movement of a cell in response to a chemical gradient.
**Cording**: property, typical of mycobacteria, to proliferate extracellularly and present a rope-like appearance.
**Cyclic-di-GMP**: second messenger used by bacteria in signal transduction.
**Emergency granulopoiesis**: hematopoietic stem cell-mediated increase of neutrophils in response to stress.
**Granuloma**: organized collection of immune cells, especially macrophages, to compartmentalize mycobacterial infection.
**Hematopoietic stem and progenitor cell (HSPC)**: pluripotent cell type that can differentiate into all types of mature blood cells.
**Inflammasome**: multisubunit protein complex assembled in response to infection via activation of caspase 1, leading to maturation of IL-1β and pyroptotic cell death.
**Persister**: bacterium tolerant to an antibiotic or an immune response.
**Primitive macrophage**: macrophage developed during embryogenesis before the emergence of pluripotent hematopoietic stem cells.
**Prostaglandin**: lipid mediator involved in the modulation of inflammation.
**Septicemia**: infection that has disseminated to the blood.
**Septin cage**: higher-order assembly of the septin cytoskeleton to entrap cytosolic bacteria and restrict proliferation.
**Type 3 secretion system (T3SS)**: molecular syringe used by Gram-negative bacteria to inject effector proteins into host cells for subversion of cellular processes.
**Zoonosis**: infectious disease transmitted to humans by contact with infected animals.
